# An Isoprenylation and Palmitoylation Motif Promotes Intraluminal Vesicle Delivery of Proteins in Cells from Distant Species

**DOI:** 10.1371/journal.pone.0107190

**Published:** 2014-09-10

**Authors:** Clara L. Oeste, Mario Pinar, Kay O. Schink, Javier Martínez-Turrión, Harald Stenmark, Miguel A. Peñalva, Dolores Pérez-Sala

**Affiliations:** 1 Department of Chemical and Physical Biology, Centro de Investigaciones Biológicas, Consejo Superior de Investigaciones Científicas, Madrid, Spain; 2 Department of Cellular and Molecular Biology, Centro de Investigaciones Biológicas, Consejo Superior de Investigaciones Científicas, Madrid, Spain; 3 Centre for Cancer Biomedicine, Faculty of Medicine, Oslo University Hospital, Oslo, Norway; NHLBI, NIH, United States of America

## Abstract

The C-terminal ends of small GTPases contain hypervariable sequences which may be posttranslationally modified by defined lipid moieties. The diverse structural motifs generated direct proteins towards specific cellular membranes or organelles. However, knowledge on the factors that determine these selective associations is limited. Here we show, using advanced microscopy, that the isoprenylation and palmitoylation motif of human RhoB (–CINCCKVL) targets chimeric proteins to intraluminal vesicles of endolysosomes in human cells, displaying preferential co-localization with components of the late endocytic pathway. Moreover, this distribution is conserved in distant species, including cells from amphibians, insects and fungi. Blocking lipidic modifications results in accumulation of CINCCKVL chimeras in the cytosol, from where they can reach endolysosomes upon release of this block. Remarkably, CINCCKVL constructs are sorted to intraluminal vesicles in a cholesterol-dependent process. In the lower species, neither the C-terminal sequence of RhoB, nor the endosomal distribution of its homologs are conserved; in spite of this, CINCCKVL constructs also reach endolysosomes in *Xenopus laevis* and insect cells. Strikingly, this behavior is prominent in the filamentous ascomycete fungus *Aspergillus nidulans*, in which GFP-CINCCKVL is sorted into endosomes and vacuoles in a lipidation-dependent manner and allows monitoring endosomal movement in live fungi. In summary, the isoprenylated and palmitoylated CINCCKVL sequence constitutes a specific structure which delineates an endolysosomal sorting strategy operative in phylogenetically diverse organisms.

## Introduction

Regulation of vesicular traffic is a key process for many cellular functions. Signal transduction by membrane receptors, protein delivery to specific compartments, membrane repair and exosome shedding or autophagy, among others, depend on compartmentalized traffic of molecules within the cell. Intracellular vesicles constitute a yet not fully characterized array of dynamic membranous compartments which are in continuous evolution and transformation, resulting in the acquisition of a specific protein and lipid composition [1], [2]. These components can regulate the fate of proteins by sorting them through different types of intracellular vesicles that can come from or recycle back to the plasma membrane, send proteins along the secretory pathway from the Golgi or travel along trafficking routes through the late endosomal pathway to the lysosome to be degraded, among many other processes.

The endolysosomal pathway is subjected to a complex regulation by families of proteins or multiprotein structures which act in a concerted manner. Indeed, small GTPases of the Rab family play a fundamental role in endosome biogenesis and traffic, and undergo the so-called “Rab conversion”, in which Rab5 from early endosomes is substituted by Rab7 at late endosomes, thus allowing progression of endosome maturation [3]. Incorporation of cytosolic proteins into this pathway is believed to occur through various mechanisms by recognizing target sequences or modifications that ultimately may trigger association with or internalization into endolysosomal vesicles. The endosomal sorting complexes required for transport (ESCRTs) constitute a well-established system of protein “machines” that work sequentially to induce the endosomal delivery of target proteins. Particular components of ESCRTs recognize ubiquitinated proteins, induce membrane invagination and promote formation of multivesicular bodies (MVB), into which proteins are sorted [4]. Other means of lysosomal sorting include modification of e.g. soluble acid hydrolases with mannose 6-phosphate (M6P) and recognition by M6P receptors in the Golgi apparatus prior to endo-lysosomal trafficking, or M6P-independent sorting by association with proteins such as sortilin or LIMP-2 [5], [6]. In the case of lysosomal transmembrane proteins, specific motifs such as dileucine or tyrosine-containing sequences can interact with clathrin-associated proteins and trigger lysosomal localization [7]. Furthermore, modifications such as phosphorylation and acylation of proteins at particular residues can also serve as vesicle-specific membrane anchors for peripheral membrane proteins [8].

One of several types of acylation, thioesterification of a palmitate molecule (C16∶0) occurs at specific cysteine residues in a reversible manner and allows proteins to cycle between different subcellular localizations. In particular, palmitoylation is important for the sorting and localization of numerous integral membrane proteins such as the M6P receptor, tetraspanins (e.g. CD9, CD63 or CD81) or synaptotagmins [9]–[11]. Small GTPases of the Ras superfamily are isoprenylated at their C-termini. For several members of this family, including H-Ras, RhoB or TC10, isoprenylation is required for the subsequent palmitoylation of nearby cysteines, which allows their association with the plasma membrane or specific endolysosomal vesicles [12]–[15]. Thus, certain protein sequences, as well as specific types of protein posttranslational modifications play a key role in targeting proteins to membranes with subtle differences in protein and/or lipid composition.

The C-terminal sequence of RhoB (CINCCKVL) is palmitoylated and isoprenylated, and these modifications are required for its localization at the endolysosomal pathway [16]. Moreover, we have previously shown that this sequence directs the rapid lysosomal degradation of chimeric proteins in bovine aortic endothelial cells (BAEC) [16]. Here we have fused the CINCCKVL sequence (or “–8”) to fluorescent proteins to explore its potential to promote endolysosomal localization in cells from different species. From our studies, CINCCKVL chimeras step forth as tunable targeting sequences which depend on posttranslational lipid modifications that can be regulated pharmacologically to finely modulate subcellular localization. Our results show that endolysosomal sorting of the chimeric proteins ending in the “–8” sequence operates in diverse cell types and is preserved in organisms as distant as fungi and humans.

## Materials and Methods

### Ethics statement

The study was conducted according to the Declaration of Helsinki principles and was approved by the Commission of Bioethics and Biosafety of Centro de Investigaciones Biológicas (Madrid, Spain) and by the Bioethics Committee of Consejo Superior de Investigaciones Científicas (Spain). All cell types used were from commercial sources (see below) or from established Cell Repositories (NIGMS Human Genetic Cell Repository at the Coriell Institute for Medical Research, Camden, NJ).

### Materials

Cell culture media RPMI1640, DMEM, TC100 and supplements (Newborn calf serum, penicillin/streptomycin, glutamine, trypsin-EDTA) were from Gibco Life Technologies (Paisley, UK). NCTC 109 medium and chloroquine were from Sigma (Steinheim, Germany). Foetal bovine serum (FBS) was from Lonza Inc. (Walkersville, MD, USA). Lipofectamine 2000 and Lysotracker Red (LTR) were from Life Technologies (Carlsbad, CA, USA). U18666A was from Merck4Biosciences (Madrid, Spain). Anti-GFP was from Roche Diagnostics GmbH (Mannheim, Germany). Anti-Hsp90 (sc-7947), anti-RhoGDI (sc-360), and anti-vimentin (sc-6260) were from Santa Cruz Biotechnology (Santa Cruz, CA, USA). Anti-mouse Igs were from Dako (Copenhagen, Denmark). Anti-giantin was from Covance (Princeton, NJ, USA).

### Plasmids

The following constructs were generous gifts: GFP-Rab7, from Prof. C. Bucci (University of Copenhagen); Lamp1-GFP, from Prof. J. Lippincott-Schwartz (NIH); GFP-Rab5, from Prof. J. Bonifacino (NIH). GFP-Rab11 was from Genecopoeia. The constructs GFP-8 (GFP-CINCCKVL), GFP-HA-RhoB and tRFP-T-HA-RhoB have been previously described [14]. The plasmid tRFP-T-8 containing a S162T mutation to improve photostability [17] was generated from tRFP-8 [16] by PCR, as previously described [14]. Dendra-8 (Dendra2-CINCCKVL) was generated from pDendra2-C (Clontech) by PCR, analogously to GFP-8, as described [16]. Briefly, the cDNA of Dendra-8 was generated by PCR using pDendra2-C as template and primers: forward, 5′-GCGCTAGCTCGAGGTACCGC-3′, and reverse, 5′-CCGAGCTCTAGAGGACCTTGCAACAGTTGATACACCACACCTGGCTGG-3′. The product was cloned in the NheI-SacI sites of pDendra2-C. The constructs GFP-CINCSKVL (GFP-8-C244S, isoprenylation defective GFP-8 mutant), GFP-SINSCKVL (GFP-8-C240, 243S, palmitoylation defective GFP-8 mutant) and the corresponding tRFP-T-8 mutants tRFP-T-CINCSKVL (tRFP-T-8-C242S) and tRFP-T-SINSCKVL (tRFP-T-8-C238, 241S), as well as the vector GFP-HA-RhoB X (expressing RhoB from *Xenopus laevis*, NCBI Acc. No.: NM_001096461.1), were generated by Genewiz Inc. (South Plainfield, NJ) by oligonucleotide synthesis and cloning into the parent vectors. The plasmid p1902 encoding Osmani GFP for fungi transformation has been previously described [18]. The vectors encoding GFP-8 and its C239, 242S (palmitoylation deficient) mutant for expression in fungi were constructed by gene synthesis and cloned into the HindIII-XmaI sites of p1902 (Genewiz).

### Cell culture and transfections

The cell line [AG09309] (primary fibroblasts from control subjects) was obtained from the NIGMS Human Genetic Cell Repository (http://ccr.coriell.org/Sections/Collections/NIGMS/?SsId=8) at the Coriell Institute for Medical Research (Camden, NJ) and was cultured according to the instructions provided. BAEC from Clonetics were cultured in RPMI1640 supplemented with 10% (vol/vol) newborn calf serum, and antibiotics (100 U/ml penicillin, 100 µg/ml streptomycin). HeLa adenocarcinoma cells from ATCC were cultured in DMEM supplemented with serum and antibiotics. *Xenopus laevis* A6 cells were obtained from Sigma and cultured at 28°C in NCTC 109 medium supplemented with 15% distilled H_2_O, 2 mM glutamine, 10% FBS and antibiotics. High Five cells, derived from ovarian cells of the cabbage looper, *Trichoplusia ni*, were from Invitrogen. These cells were cultured at 28°C in TC100 medium supplemented with 10% FBS, penicillin-streptomycin as above and 10 µg/ml gentamycin. Transfection of cells at 80% confluence was performed with Lipofectamine2000 following the instructions of the manufacturer, using 1 µg of DNA and 3 µl (single transfections) or 4.5 µl (double transfections) of Lipofectamine per p35 dish. Unless otherwise indicated, after transfection, cells were allowed to recover for 24 h in complete medium before treatment with the indicated agents in the absence of serum. Imaging of live cells was performed 48 h after transfection to minimize its effects.

### *Aspergillus nidulans* culture

*Aspergillus nidulans* was cultured on complete medium (MCA) or synthetic complete medium (SC) containing 1% glucose and 5 mM ammonium tartrate (i.e. 10 mM NH^4+^) as carbon and nitrogen sources, respectively. GFP-8 and GFP-8-C239S, C242S were expressed under the control of the gpdA^mini^ promoter, using a single-copy integration construct targeted to *pyroA*, as described previously [18]. Strains used in this work: MAD4688: wA2; pyroA4::[pyroA*-gpdA^mini^::GFP-8-C239S, C242S]; pantoB100 and MAD4689: wA2; pyroA4::[pyroA*-gpdA^mini^::GFP-8]; pantoB100.

### Immunofluorescence

For immunofluorescence with anti-giantin antibody, cells were fixed in 4% paraformaldehyde for 20 min at r.t., washed twice in PBS and permeabilized with 0.05% saponin in PEM buffer (80 mM PIPES, 5 mM EGTA, 1 mM MgCl_2 _pH 6.8) for 5 min. Incubation with anti-giantin at 1∶500 dilution was carried out in PBS containing 0.05% saponin for 1 h. After PBS washes, cells were incubated with anti-rabbit-Alexa 568 (Invitrogen) at 1∶200 in 1% BSA in PBS for a further hour.

### Microscopy

For live cell visualization, cells were cultured on glass-bottom dishes (Mattek Corp., Ashland, MA), transfected with fluorescent constructs, treated with the indicated agents and visualized directly on a confocal microscope (LEICA DMRE2 or LEICA SP5 Heidelberg, Germany). Unless otherwise stated, images shown are single channels or overlays of single Z-sections for co-localization visualization. All experiments were repeated at least three times and representative results are shown. Bars represent 20 µm unless otherwise indicated.

*In vivo* superresolution 3D structured illumination microscopy (SIM) imaging was performed on a Deltavision OMX V4 system (Applied Precision, a GE healthcare company, Issaqua, WA) equipped with an Olympus 60x NA 1.42 objective, cooled sCMOS cameras and 405, 488, 568 and 642 nm diode lasers. Z-stacks covering the whole cell were recorded with a Z-spacing of 125 nm. A total of 15 raw images (5 phases, 3 rotations) per plane were collected. Reconstruction and alignment of these raw images was performed using Softworx software. (Applied Precision, a GE healthcare company, Issaqua, WA).

Dendra-8 photoswitching and isoprenylation inhibition-and-release experiments were performed on transfected BAEC on glass-bottom dishes. The transfection medium was removed after 5 hours and replaced with serum-free medium with or without 10 µM simvastatin. Cells were observed 24 hours post-treatment on a confocal microscope (LEICA SP2) and photoswitching was performed by exposure to 405 nm UV light. The resulting green-to-red conversion was recorded in both channels. Simvastatin was either added or removed to the corresponding dishes in serum-free medium and photoconverted, red Dendra-8 was tracked 24 hours later by confocal microscopy.

For microscopy experiments in *Aspergillus*, hyphae were cultured in Lab-Tek chambers (Thermo Fischer Scientific, 115411; 0.3 ml of medium per well) at 25–28°C in pH 6.5 ‘watch minimal medium’ (WMM) containing 100 mM sodium acetate and 5 mM ammonium tartrate as carbon and nitrogen sources, respectively. Hyphae were visualized on an inverted fluorescence microscope (Leica DMI6000B) equipped with an EL6000 external light source with metal halide lamp epifluorescence excitation, driven by Metamorph software (Molecular Dynamics) and coupled to a CCD camera (ORCA ER-II; Hamamatsu). Vacuoles were detected with CMAC (7-amino-4-chloromethylcoumarin, CellTracker Blue; Invitrogen C-2110).

### Image analysis

Co-localization analysis was performed with LAS-AF software from Leica on single z-sections of images to obtain co-localization rates (expressed as percentages) and Pearson coefficients (coefficient *r*×100). At least 30 cells were analysed per experimental condition. Results are presented as average co-localization rates or Pearson coefficients ± standard error of mean (SEM) as indicated. Average values were compared by Student’s t-test for unpaired observations.

### Western blot

SDS-PAGE and Western blot analysis of HeLa cells were performed as described [16], [19], using ECL detection (GE Healthcare, Buckinghamshire, UK). For analysis of cell fractions, S100 and P100 fractions were obtained by centrifugation of total cell lysates at 100,000×g for 1 h at 4°C, essentially as previously described [20].

## Results

### Fluorescent CINCCKVL constructs localize at endolysosomal compartments in human cells

**Figure 1:**
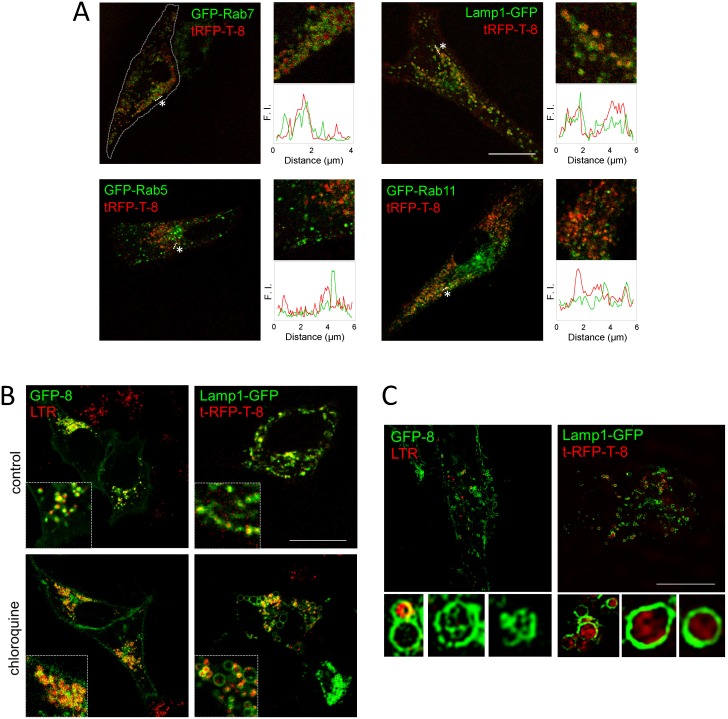
Localization of CINCCKVL chimeras in human cells. (A) Human fibroblasts were transfected with the indicated constructs and observed live by confocal microscopy after 16 h in serum-depleted medium. To the right of every condition, zoom-ins of original pictures and fluorescence intensity profiles along a section (see dotted lines marked by asterisks) are shown. The magnification used for zoom-ins is seven-fold with respect to the corresponding full-sized images. F.I., fluorescence intensity. (B) HeLa cells were transiently transfected with the indicated constructs and treated with 25 nM LTR for 15 min, where indicated, prior to live confocal microscopy observation. Bottom panels show cells that were treated with 10 µM chloroquine 24 h post-transfection for a further 24 h. Insets show details of the pictured cells. (C) Cells transfected as in (B) were visualized live by super-resolution microscopy (SIM). Insets show individual MVB and their ILV decorated with CINCCKVL fluorescent proteins. Scale bar, 20 µm.

To assess the ability of the CINCCKVL motif to induce endolysosomal localization in distant species, we first assessed the distribution of fluorescent CINCCKVL chimeric proteins in primary human fibroblasts, a species in which these constructs have not been characterized. Therefore, we co-transfected tRFP-T-CINCCKVL (tRFP-T-8) with fluorescent constructs of known components of various endosomal compartments. Fusion proteins of Rab family GTPases are commonly used as markers of particular endosomal vesicles, i.e. fluorescent constructs of Rab5 or Rab7 for early and late endosomes, respectively. As can be observed in Figure 1A, tRFP-T-8 localized inside vesicles delimited by GFP-Rab7 and the late endosome-lysosomal marker Lamp1-GFP. Under our conditions, tRFP-T-8 showed partial co-localization with GFP-Rab11, which typically localizes to recycling endosomes [21], and more limited co-localization with a fluorescent construct of Rab5 [22]. Thus, the tRFP-T-8 construct displays a selective localization at late endosomes-lysosomes in this cell type (Fig. 1A, see profiles).

In the widely used human cell line from cervical adenocarcinoma HeLa, CINCCKVL-chimeric proteins showed substantial co-localization with LTR (GFP-8/LTR co-localization rate: 62.6±2.5%) and tRFP-T-8 localized inside Lamp-1-GFP-positive vesicles which, as described above, became dilated when treated with cloroquine (Fig. 1B). GFP-8 also co-localized extensively with a fluorescent construct of CD63, a component of MVB that is used as a marker of intraluminal vesicles (ILV) (Fig. S1A). This prominent co-localization was confirmed in BAEC (Fig. S1B). Consistent with this and taking advantage of the higher localization precision obtained by *in vivo* super-resolution structural illumination microscopy (SIM), we further demonstrated the presence of both GFP-8 and tRFP-T-8 in the ILV of MVB (Fig. 1C). This suggests that this simple amino acid sequence can carry proteins to specific domains in endolysosomes, some of which are ultimately internalized to form vesicles inside MVBs. Inside these compartments, whereas tRFP-T-8 showed a more diffuse pattern, GFP-8 outlined the inner membranes, which could be attributed to the decrease in GFP fluorescence occurring in acidic pH environments [23].

### Lipidation of CINCCKVL acts as an endolysosomal targeting switch

**Figure 2:**
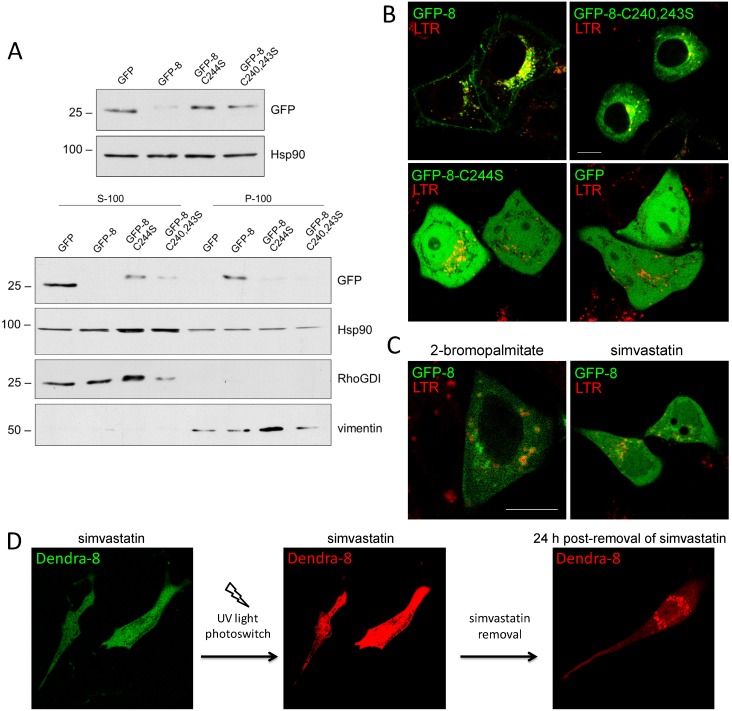
Endolysosomal localization of CINCCKVL-chimeric proteins depends on posttranslational processing. (A) HeLa cells were transiently transfected with the indicated constructs and 24 h later they were cultured in serum-free medium for another 24 h. Cell lysis and fractionation were achieved as described in the Experimental section. Upper panels show the amount of the constructs in total cell lysates and lower panels depict the levels of the constructs in S100 (soluble) and P100 (particulate) fractions, assessed by western blot with an anti-GFP antibody. Hsp90 was used as a loading control, RhoGDI as a marker of the soluble fraction and vimentin as a particulate fraction marker. Results are representative from three experiments with virtually identical results. The positions of the 25 and 100 kDa markers are shown for reference. (B) HeLa cells were transfected with GFP-8, its palmitoylation defective mutant (GFP-8-C240, 243S), its isoprenylation defective mutant (GFP-8-C244S) or GFP, as indicated, and stained with LTR prior to live confocal microscopy imaging. (C) GFP-8-transfected HeLa cells were treated with 20 µM 2-bromopalmitate for 6 h or 10 µM simvastatin for 24 h in serum-free medium and stained with LTR before live observation by confocal microscopy. (D) BAEC were transfected with Dendra-8 and treated with simvastatin for 24 h (left panel) prior to eliciting a green-to-red photoswitch using UV light (middle panel). Immediately after photoswitching simvastatin was removed and the localization of the red, photoconverted protein was assessed 24 h later (right panel). Scale bar, 10 µm.

In RhoB, the CINCCKVL motif is subject to posttranslational processing at its cysteine residues by irreversible binding of an isoprenoid to its last cysteine and reversible palmitoylation of its two remaining cysteines. To explore the importance of these modifications in endolysosomal localization of CINCCKVL-chimeric proteins, we generated constructs in which the lipidation cysteines have been mutated. Total lysates from cells transfected with these constructs are shown in Figure 2A, upper panels. The degree of membrane association of the different constructs was assessed by subcellular fractionation into S100 (soluble) and P100 (membrane) fractions, followed by western blot (Fig. 2A, lower panels). Whereas GFP was fully soluble, GFP-8 was totally membrane bound. In turn, the isoprenylation and the palmitoylation defective mutants (GFP-8-C244S and GFP-8-C241, 243S, respectively) showed a predominant localization in the soluble fraction with a marginal amount appearing in P100 fractions.

Membrane localization of the different constructs was also assessed by confocal microscopy (Fig. 2B). Mutation of the palmitoylation cysteines in GFP-8 altered its typical localization, leading to the cytoplasmic distribution of the non-palmitoylatable construct (GFP-8-C241, 243S), which excluded the nucleus. This pattern has been observed for constructs that can be isoprenylated but not palmitoylated, such as GFP bearing only a CAAX motif [16]. In some cells, certain cytoplasmic accumulations of the GFP-8-C241, 243S construct were observed that corresponded to its presence at the Golgi, as indicated by their co-localization with the Golgi component giantin (Fig. S2). In contrast, mutation of the CAAX box cysteine (GFP-8-C244S) led to a diffuse distribution throughout the cytoplasm and nucleus, excluding some organelles (Fig. 2B). This pattern was indistinguishable from that of GFP and is typical of non-lipidated constructs, thus indicating that isoprenylation of GFP-8 is required for subsequent palmitoylation [24]. Similar results were obtained with tRFP-T-8 palmitoylation or isoprenylation-defective mutants (results not shown). Importantly, pharmacological inhibition of palmitoylation by means of treatment with 2-bromopalmitate or of isoprenylation (and subsequent palmitoylation) with simvastatin, resulted in a cellular distribution of GFP-8 analogous to that of the corresponding palmitoylation or isoprenylation-defective mutants (Fig. 2C). Therefore, association of CINCCKVL chimeras with membranes can be modulated pharmacologically by blocking lipidation of its cysteine residues. We took advantage of this feature to address the key issue of whether a non-lipidated, cytosolic pool of CINCCKVL protein can undergo lipidation to be sorted to the aforementioned membranes or whether sorting must occur on newly synthesized proteins. Using BAEC, which present low cytotoxicity upon treatment with simvastatin, and Dendra2, a protein capable of photoswitching from green to red fluorescence emission after UV light exposure, we set up a simvastatin-based approach to follow specific pools of a Dendra2-CINCCKVL fusion protein (Dendra-8, Fig. 2D). Treatment with simvastatin blocked processing of Dendra-8 rendering a pool of fully cytosolic green construct. This pool was photoswitched obtaining a soluble, non-isoprenylated red fluorescent CINCCKVL construct to follow. Releasing the isoprenylation inhibition by removal of simvastatin allowed endolysosomal targeting of the previously diffuse red construct, clearly demonstrating that preexisting CINCCKVL-chimeric proteins may undergo sorting from a cytosolic localization, depending on isoprenoid availability (Fig. 2D). The observed behavior establishes this sequence as a tunable targeting motif in live cells.

### Impaired cholesterol dynamics alters sorting of CINCCKVL chimeras

**Figure 3:**
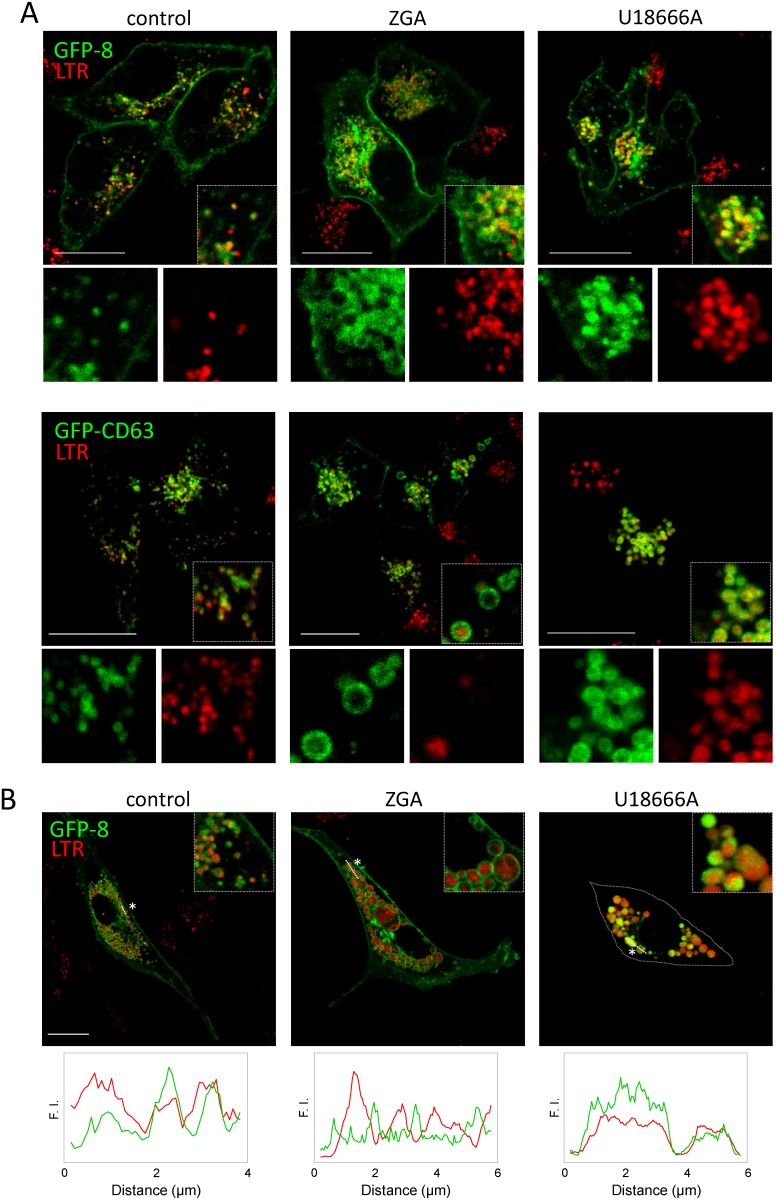
Effects of agents modulating cholesterol synthesis and traffic on the distribution of CINCCKVL constructs. (A) HeLa cells were transfected with GFP-8 (upper panels) or GFP-CD63 (lower panels). 24 h later cells were treated with 100 µM ZGA or 10 µM U18666A for 24 h and stained with LTR as above. Insets show enlarged areas of interest. The single channels corresponding to the areas in insets are shown below each image. (B) BAEC were transfected with GFP-8 and treated with ZGA or U18666A as described in (A). Insets show enlarged areas of interest and lower panels depict fluorescence intensity profiles along a section (see dotted lines marked by asterisks).

Specific combinations of lipidic membrane anchors, such as that conferred by the CINCCKVL sequence, may promote protein association with membrane domains of a particular composition [15]. To address this issue, here we have tackled cholesterol availability and traffic in the HeLa cell model (Fig. 3). Remarkably, inhibiting cholesterol biosynthesis with the squalene synthase inhibitor zaragozic acid (ZGA) induced the appearance of enlarged endolysosomes showing a diffuse localization of GFP-8, which in some of them appeared retained at the edge (Fig. 3A, upper panels). Some of these structures displayed a fainter or patchy LTR staining. In sharp contrast, U18666A, an agent that causes accumulation of cholesterol and of the endosomal lipid 2,2′-dioleoyl lysobisphosphatidic acid (LBPA), also known as bis(monoacylglycero)phosphate (BMP) in MVB [25], induced the formation of dense MVB containing GFP-8, as previously observed in BAEC [14]. Interestingly, GFP-CD63, a marker of MVB intraluminal structures, suffered alterations similar to those of GFP-8 in response to ZGA and U18666A (Fig. 3A, lower panels). Moreover, CD63 markedly co-localized with CINCCKVL constructs at the altered endolysosomes under all experimental conditions (Fig. S1C). In order to substantiate the importance of cholesterol availability for GFP-8 sorting, we treated BAEC with ZGA. Inhibition of cholesterol biosynthesis elicited the appearance of dilated MVB showing a peripheral distribution of GFP-8 (Fig. 3B) and CD63 (Fig. S1D). This cell type presents large endosomes, so that the differences between the alterations induced by ZGA and U18666A (shown here for reference) were more obvious, clearly showing the reduction of intraluminal content in ZGA-treated cells (Fig. 3B, see profiles). Taken together these results suggest that the specific modification of CINCCKVL chimeras induces their association with defined membrane domains, in a manner dependent on the presence or distribution of cholesterol, which appears to be required for their sorting.

### Localization of CINCCKVL chimeric proteins in cells from non-mammalian species

**Figure 4:**
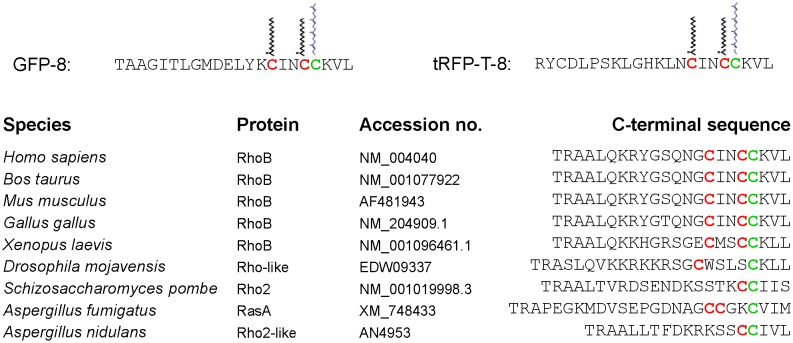
C-terminal sequences of CINCCKVL-chimeric proteins, RhoB homologs and related proteins from diverse species. The C-terminal sequences of GFP-8 and tRFP-T-8 are shown on top, together with a schematic view of the lipidic modifications; palmitates are shown in black and the geranylgeranyl moiety in blue. The Pubmed accession numbers of genes coding for proteins bearing C-terminal sequences similar to that of RhoB are shown in the lower panel. Potential sites for palmitoylation are shown in red and the isoprenylation cysteine in green.

The C-terminal sequence of RhoB is unique for this GTPase and it is conserved in mammalian species and in birds (Fig. 4). In lower species, the RhoB sequence displays a higher degree of variability, and in some cases a *bona fide* homolog of RhoB has not been identified (Fig. 4). Although several proteins can be found which possess CAAX boxes and potential sites for palmitoylation, the composition and/or spacing of these residues is not conserved with respect to the CINCCKVL sequence. Therefore, we wanted to assess whether CINCCKVL constructs would confer endolysosomal localization in more distant non-mammalian species.

**Figure 5:**
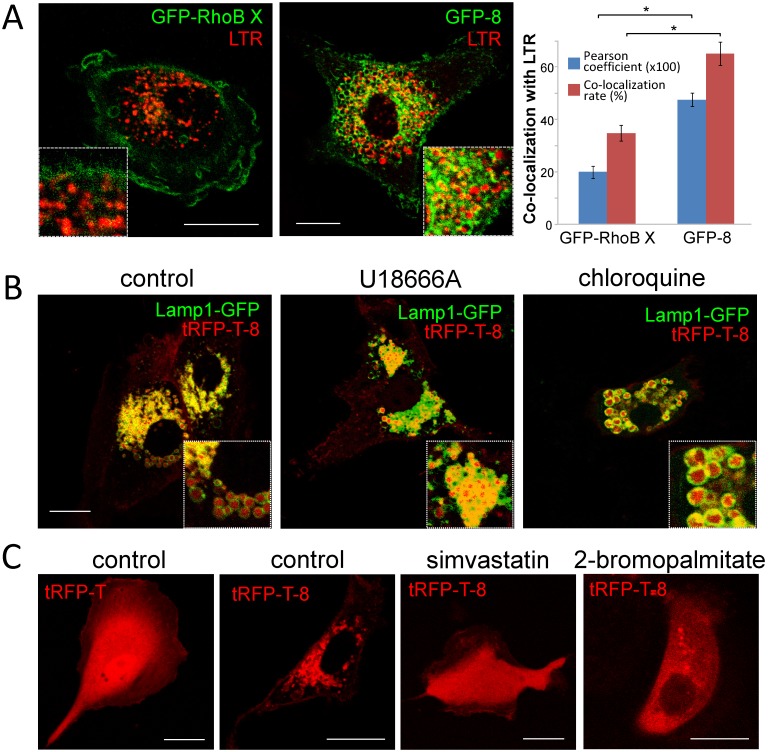
Localization of RhoB-related proteins in amphibian cells. (A) *Xenopus laevis* RhoB (GFP-RhoB X) or GFP-8 were expressed in *Xenopus laevis* A6 cells and acidic compartments were stained with LTR. The right panel depicts the extent of co-localization of GFP and LTR signals shown as Pearson coefficients (x100) or co-localization rates (in percentages). Results are average values of at least 30 cells per condition ± standard error of mean (SEM). *p<1×10^−6^ *vs* GFP-8 by Student’s t-test. (B) Effect of agents altering lysosomal function on the distribution of tRFP-T-8. *Xenopus* A6 cells were co-transfected with Lamp1-GFP to mark lysosomes and tRFP-T-8, and treated with 10 µM U18666A or 10 µM chloroquine, as indicated. (C) Dependence of tRFP-T-8 localization on posttranslational modification in *Xenopus* cells. A6 cells were transfected with tRFP-T or tRFP-T-8 and treated with 10 µM simvastatin or 20 µM 2-bromopalmitate and imaged live by confocal microscopy.

The *Xenopus laevis* RhoB protein C-terminus contains one isoprenylation and two putative palmitoylation cysteine residues (Fig. 4). However, the composition of this sequence clearly differs from that of human RhoB in the presence of a basic amino acid cluster at position 181–183 (KKH), and in the amino acids located between the two palmitoylation cysteines (MS). These features are analogous to motifs present in the C-terminal segments of TC10 (KKH), an endosomal RhoB-related GTPase, and H-Ras (MS), which preferentially localize to recycling compartments and to the plasma membrane, respectively [12], [14]. Consistent with this, we observed that GFP-tagged *Xenopus laevis* RhoB (GFP-RhoB X) was mainly localized at the plasma membrane in *Xenopus* A6 cells, and showed limited co-localization with LTR (Fig. 5A, left panel). In contrast, transfection of CINCCKVL chimeric proteins in *Xenopus* A6 cells resulted in a clear endolysosomal localization of the constructs, as revealed by the disposition of GFP-8 at LTR-positive compartments (Fig. 5A, middle panel), showing substantial co-localization with this lysosomal marker (Fig. 5A, right panel) and the presence of tRFP-T-8 inside vesicles delimited by Lamp1-GFP (Fig. 5B). Interestingly, human RhoB but not *Xenopus* RhoB fluorescent constructs extensively co-localized with CINCCKVL constructs in A6 cells (Fig. S3), thus stressing the importance of the differences in the sequence of RhoB from various species for targeting of the full-length protein. This also indicates that, although the RhoB C-terminal sequence is not fully conserved in *Xenopus laevis*, the mechanisms targeting CINCCKVL-chimeric proteins to endolysosomes are operative in this cell type.

We next assessed the distribution of these constructs in response to agents which disrupt endolysosomal dynamics and cholesterol traffic [16]. Treatment of A6 cells with U18666A led to a dramatic compaction of endolysomes, which accumulated tRFP-T-8 (Fig. 5B), and chloroquine caused accumulation of tRFP-T-8 inside enlarged endolysomes (Fig. 5B). Moreover, we confirmed that in these cells, targeting of CINCCKVL chimeric proteins was also dependent on their posttranslational processing by isoprenylation and palmitoylation. As shown in Fig. 5C, treatment with simvastatin abolished the distribution of tRFP-T-8 in endosomal vesicles and led to a diffuse distribution throughout the cytosol and the nucleus, indistinguishable from that of tRFP-T, thus confirming the importance of isoprenylation for tRFP-T-8 targeting in this cell type, as well. In contrast, inhibition of palmitoylation with 2-BP elicited the appearance of the protein in the cytosol, excluding the nucleus, as shown above for GFP-8 in Hela cells. Similar experiments were routinely carried out in other cell types used in this work and yielded analogous results (data not shown).

**Figure 6:**
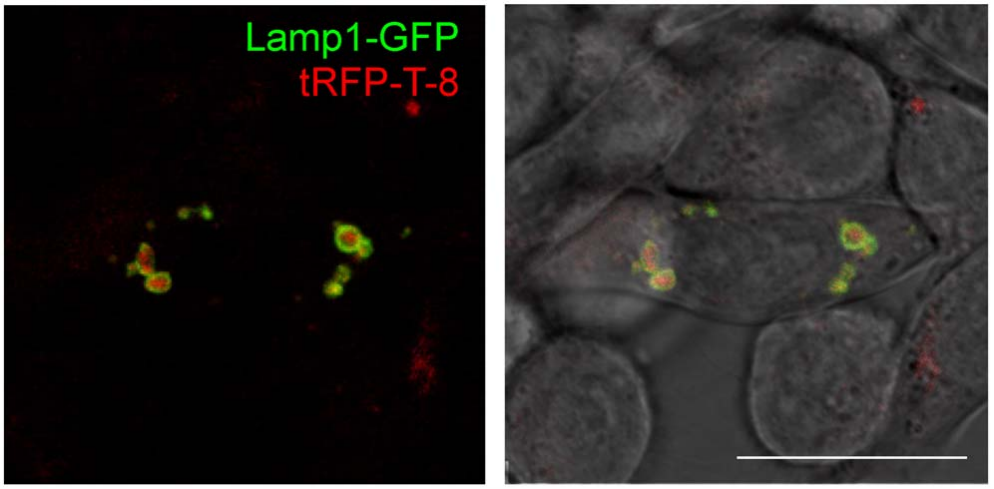
Localization of tRFP-T-8 in High Five insect cells. High Five cells were transfected with Lamp1-GFP and tRFP-T-8 and live cells were visualized by confocal microscopy. The overlays of single fluorescent z-sections alone and with the Differential Interference Contrast image are shown.

In order to ascertain the robustness of the CINCCKVL sequence as a determinant for endolysosomal localization, we went on to explore its localization in a cell line of an invertebrate. In High Five insect cells, tRFP-T-8 was fully localized inside vesicles delimited by Lamp1-GFP, thus suggesting its lysosomal targeting (Fig. 6). Interestingly, in insect cells the homology of Rho-related proteins with RhoB is more distant, both in terms of the number of cysteines located at the C-terminus and the nature of the surrounding amino acids (Fig. 4). Nevertheless, the sequence derived from the mammalian protein still promotes localization in lysosomal compartments.

### Tracking endosomes with GFP-CINCCKVL in *Aspergillus nidulans*

**Figure 7:**
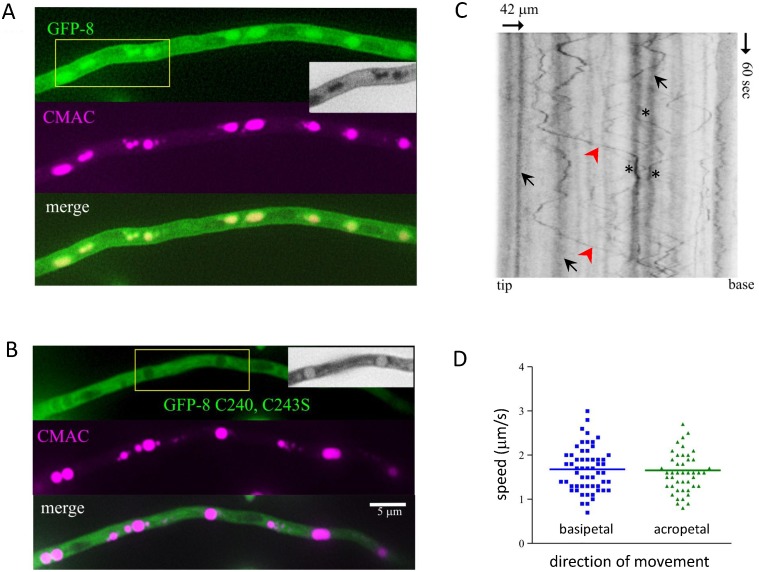
Localization of GFP-8 in *Aspergillus nidulans*. Strains of *Aspergillus nidulans* expressing GFP-8 or its palmitoylation deficient mutant (GFP-8-C240, 243S) were imaged as described in Methods. (A and B) show co-localization of the constructs with the vacuole marker, CMAC. Insets show grayscale images for better contrast. (C) Kymograph showing the movements of various GFP-8-positive compartments. Rapidly moving endosomes (likely corresponding to early endosomes) are marked by arrowheads (red), static vesicles (likely corresponding to vacuoles) are marked by arrows and potential points of contact between endosomes and vacuoles are depicted by asterisks. (D) Graph representing velocities of individual endosomes.

Palmitoylation plays a key role in protein localization and sorting in lower eukaryotic organisms such as fungi [26]. Indeed, yeast have been seminal for the identification and characterization of the isoprenylation and palmitoylation machinery. The fungus *Aspergillus nidulans* expresses a potential Rho-related protein bearing one putative site for palmitoylation adjacent to the isoprenylation cysteine (Fig. 4). However, in this organism, the –CINCCKVL sequence is not conserved. In *Aspergillus nidulans* most GFP-8 fluorescence clearly localized in late endosomes and vacuoles, where it co-localized with 7-amino-4-chloromethyl-coumarin (CMAC), a marker for hydrolase-containing acidic organelles (Fig. 7A) [27]. In addition, GFP-8 fluorescence was detected at endosomes showing bidirectional movement (see Video S1), which is dependent on microtubules [27]. In this model, GFP-8 also decorated the plasma membrane (Fig. 7A, inset) and septa (not shown). GFP-8 localization was dependent on its posttranslational modification since a palmitoylation-deficient mutant, GFP-8-C240, 243S, was fully cytosolic, excluding plasma membrane, septa, vacuoles and nuclei (Fig. 7B). Therefore, analogously to the observations in cells from higher organisms, the isoprenylation motif was not sufficient to promote the specific endolysosomal localization of this construct, although it led to nuclear exclusion. Nevertheless, the distribution of the GFP-8-C240, 243S construct was distinct from that of GFP, which, as observed in mammalian cells, showed a diffuse pattern throughout the nucleus and cytosol (not shown).

*Aspergillus nidulans* constitutes a well characterized model for monitorization of the endocytic process. Fluorescent constructs of endosomal proteins allow following the movement of these organelles by time-lapse microscopy and representation of their trajectories as kymographs to calculate endosomal speed [27]. Therefore, we took advantage of these possibilities to characterize the dynamics of GFP-8-positive compartments in more detail. Figure 7C shows a representative kymograph where nearly static traces correspond to vacuoles (arrows). In addition, the movement of various endosomes, both in basipetal and acropetal directions, can be monitored (arrowheads). Moreover, points of endosome-vacuole contact can be observed (asterisks). The velocities of the endosomes monitored are shown in Figure 7D. As can be observed, GFP-8-positive compartments show speeds ranging from less than 1 µm per second, to nearly 3 µm per second, the average velocities being practically the same for basipetal and acropetal movements. Therefore, GFP-8 marks the endolysosomal pathway in *Aspergillus nidulans*, suggesting that the targeting mechanisms for CINCCKVL constructs are also conserved in fungi.

## Discussion

The combination of several lipidic modifications, like isoprenylation and palmitoylation constitute important determinants for protein localization in specific subcellular compartments. The spacing of the lipid moieties and the nature of the non-lipidated amino acids may contribute to the generation of unique structures with affinity for membrane domains or protein partners. Here we have shown that the isoprenylation and palmitoylation motif of the GTPase RhoB constitutes one such structure *per se* and is able to determine protein sorting to lysosomal compartments in cells from distant species, including fungi and humans.

Several mechanisms for sorting to MVBs have been reported. The classical mechanism implies ubiquitination of the cargo protein and association with components of the ESCRT machinery, which, according to some models may assemble sequentially to define invagination domains leading to the formation and ultimately the release of ILV [7], [28], [29]. Although under our conditions we have not detected ubiquitination of GFP-8 (unpublished observations), this mechanism cannot be excluded since ubiquitination-independent sorting of certain cargo by ESCRT-mediated processes has also been reported [30]. Recently, a late-endosome microautophagy-like process has been described, which depends on ESCRT I and III for vesicle formation and on Hsc70 for cargo selection, and may mediate the delivery into late endosomes of various cytosolic proteins [31]. Nevertheless, this process does not seem to be involved in sorting of CINCCKVL chimeric proteins since it is disrupted by U18666A, which as shown above, causes strong accumulation of GFP-8 in MVB. In turn, although the autophagic and endolysosomal pathways may converge at several levels, we have previously observed that GFP-8 and the autophagosomal marker RFP-LC3 show different distribution patterns [32], Moreover, endolysomal targeting of CINCCKVL constructs occurs also in cells growing in complete medium, including human, *Xenopus* and insect cells (unpublished observations), thus indicating that induction of macroautophagy is not required for sorting. Lastly, in chaperone-mediated autophagy, unfolding of the protein occurs prior to entrance in the lysosome, leading to a loss of fluorescence [33]. Under our conditions, however, intra-lysosomal fluorescence of CINCCKVL-constructs is preserved, thus making this potential mechanism unlikely.

The fact that CINCCKVL proteins are targeted to human lysosomes and fungal vacuoles suggests the presence of conserved sorting mechanisms in these distant species. Indeed, extensive phylogenetic studies on Rab family proteins have unveiled the existence of more than 20 core Rab proteins in the latest eukaryotic common ancestor (LECA) which have suffered expansions *via* gene duplication or losses throughout eukaryotic evolution giving rise to modern Rabs [34], [35]. Under our conditions, the Rab proteins that co-localize to a greater extent with CINCCKVL chimeras are encompassed in a proposed late endosomal LECA Rab family (group III), making it possible that particular Rabs of this conserved group could be involved in CINCCKVL sorting. Similarly, the ESCRT protein family is also conserved throughout eukaryotes, in particular ESCRT-I, -II, -III and -III-associated proteins, though ESCRT-0 proteins appear to be specific to Opisthokonta (fungi and animals/metazoans) [36]. Interestingly, although several tetraspanin homologs have been identified in fungi, including *Aspergillus* species [37], their location and/or role in vesicular traffic have not been studied. Therefore, the species studied here may share many of the MVB sorting machineries already present in the LECA, underscoring the conserved character of the fundamental trafficking proteins involved in later steps of the endolysosomal pathway.

In addition to the roles of proteins or protein complexes, lipids with a particular structure may contribute to the sorting process by forming targeting microdomains or through the induction of curvature which facilitates membrane invagination. This is the case of the late endosomal lipid LBPA/BMP [38]. Interestingly, we have observed that pharmacological disruption of MVB lipid dynamics with U18666A traps GFP-8 in this compartment in several species, including *Xenopus laevis*. Remarkably, inhibition of cholesterol biosynthesis with ZGA led to MVB alterations consisting in dilation and retention of ILV components, including CD63 and GFP-8, at the periphery of some of these dilated compartments. Results obtained recently in mice deficient in ^24^ sterol reductase, the last enzyme of the cholesterol biosynthetic pathway, in which a reduction in MVB intraluminal material has been reported, might serve as a clue to the behavior observed here in BAEC and HeLa cells [39]. It has been reported that most of the cholesterol present in endolysosomes is contained in ILV. Therefore, it is plausible that an overall metabolic cholesterol reduction would affect ILV more directly than other compartments [40]. Conversely, treatment of cells with methyl-β-cyclodextrin, widely used to deplete plasma membrane cholesterol, did not alter GFP-8 distribution, although a non-specific alteration of cell morphology, prior to cell rounding, was noticed (unpublished observations). Taken together, our results indicate that interaction with endosomal lipids is an important determinant in GFP-8 sorting.

In all species studied, palmitoylation appears as a key element for lysosomal localization of CINCCKVL chimeras. Protein palmitoylation has been involved in protein sorting, although its effects appear to be cell-type and protein-specific. The mannose receptor N-terminus is palmitoylated and blocking this modification induces lysosomal accumulation of this protein [9]. In the case of the protease-activated receptor 1, palmitoylation allows the correct utilization of tyrosine-based sorting signals, and a palmitoylation-deficient mutant, shows increased degradation in lysosomes [41]. In contrast, the Ca^2+^ sensor synaptotagmin 7 has been reported to be targeted to lysosomes by its palmitoylation-dependent association with the tetraspanin CD63 [42], although the examples of palmitoylation-driven endolysosomal association in mammalian cells are very scarce. In yeast, the palmitoylated protein Vac8 is targeted to the vacuolar membrane, although there is no evidence for vacuolar sorting [43]. Therefore, in most cases, palmitoylation directs proteins away from lysosomes (see [44] and [16] and references therein), with only a few examples of palmitoylation-supported ILV sorting. Interestingly, as previously suggested [45], palmitoylation is also likely responsible for the lack of interaction of GFP-RhoB or GFP-8 with RhoGDI (Oeste et al., unpublished observations). Nevertheless, it would be interesting to assess whether non-palmitoylated constructs interact with RhoGDI in the cytosol.

The human RhoB sequence is not conserved in the lower species, in which its closest homologs are not endosomal proteins. Yet, CINCCKVL chimeric proteins show endolysosomal localization analogous to that found in mammalian cells. In insects, a clear homolog of RhoB has not been identified. However, several proteins exist that possess sequences for bipalmitoylation, although their potential lysosomal targeting has not been explored. In *Schizosaccharomyces pombe*, the RhoB homolog Rho2 has been reported to be mainly membrane bound and localize at the growing end(s) of the cell and the septation site [46]. Rho2 is isoprenylated and palmitoylated [26], although, as predicted from its CAAX box sequence, is farnesylated [47], whereas RhoB is mainly geranylgeranylated in cells [48]. However, when using constructs directing either farnesylation of geranylgeranylation of GFP-8, namely, GFP-CINCCKVL and GFP-CINCCLVM, we did not find significant differences in their lysosomal localization, thus indicating that the length of the isoprenoid moiety is not a critical factor (Oeste et al., unpublished observations). In addition, Rho2 only possesses one palmitoylation cysteine adjacent to the isoprenylation site. Also in yeast, there are examples of bipalmitoylated proteins, like RasA, which localizes at the plasma membrane, but appears in internal patches when palmitoylation is blocked [49]. However, in this case, the spacing of palmitates is different from that of CINCCKVL proteins (see Fig. 4). In this context, we have previously shown than the spacing of palmitates is key for fine-tuning protein localization. Moving the distal palmitoylation cysteine away from the isoprenylation site in a chimeric construct reduces its accummulation in MVB in response to alterations in lipid dynamics [14]. In *Aspergillus nidulans*, both the putative Rho2-like protein (Fig. 4) and GFP-8 would be expected to be geranylgeranylated and palmitoylated, although, to the best of our knowledge, a homolog for GGTase I has not been biochemically identified in this organism and the information available on potential geranylgeranyltransferases only stems from sequence homology analysis on database searches. It can be noted that CINCCKVL chimeras displayed some degree of plasma membrane localization, although it was dependent on the cell type under study and on the construct used, with GFP-8 decorating the plasma membrane to a higher extent than tRFP-T-8. Nevertheless, the most consistent localization of CINCCKVL chimeric proteins, when lipidated, is the endolysosomal compartment. It could be hypothesized that the specific structure formed upon CINCCKVL lipidation promotes the interaction with specific microdomains that require the presence of cholesterol. Indeed, domains rich in free cholesterol have been described in MVB of endothelial cells under particular cell culture conditions [50]. Moreover, the presence of lipid microdomains that segregate proteins in the yeast vacuole has been recently demonstrated in *Saccharomyces cerevisiae* [51].

One of the most striking conclusions of this work is that the CINCCKVL sequence takes a route to endolysosomes present in the lower species but not used, to the best of our knowledge, by their endogenous proteins. Our findings may motivate the search for proteins with analogous sequences that may be sorted through this pathway, as well as the use of cells from lower species to completely unveil the sorting mechanism. This information will allow elucidating whether this sequence has evolved in the higher species to ensure endolysosomal delivery of specific proteins. In summary, our observations support the interpretation that the structures generated by precise lipidation sequences, and in particular by the CINCCKVL motif, may specifically interact with defined membrane components for protein targeting and sorting. These findings may set the basis for exploring the sorting of lipidated sequences to specific microdomains involved in MVB biogenesis in diverse species.

## Supporting Information

Figure S1 **GFP-8 co-localization with the MVB/ILV marker, mCherry-CD63.** (A) HeLa cells were co-transfected with GFP-8 and mCherry-CD63, serum-starved for 16 h and observed by live confocal fluorescence microscopy. (B) BAEC were treated as described for HeLa. Scale bar, 20 µm. (C) HeLa cells transfected as above were treated with ZGA or U18666A as in Figure 3. (D) BAEC transfected as in (A) were treated with ZGA as in Figure 3. Insets show enlarged areas of interest. The single channels corresponding to the areas in insets are shown below each image.
(TIF)

Figure S2 **Golgi staining of cells transfected with GFP-8 and its mutants.** HeLa cells transfected with GFP, GFP-8 or the mutants featured in Figure 2 were fixed after 16 h of serum deprivation and the Golgi compartment was stained by anti-giantin immunofluorescence, as described in Materials & Methods.
(TIF)

Figure S3 **Localization of** ***Xenopus*** **or human RhoB chimeras in amphibian cells.** *Xenopus laevis* A6 cells were co-transfected with the indicated constructs and observed live by confocal microscopy after 16 h in serum-depleted medium. Insets show enlarged areas of interest.
(TIFF)

Video S1 *Aspergillus nidulans* expressing GFP-8 and showing endosome movement.
(MOV)
