# Supplementary figures and images for: An Isoprenylation and Palmitoylation Motif Promotes Intraluminal Vesicle Delivery of Proteins in Cells from Distant Species
Source: PLoS One. 2014 Sep 10;9(9):e107190. doi: 10.1371/journal.pone.0107190 (PMC4160200; doi:10.1371/journal.pone.0107190)

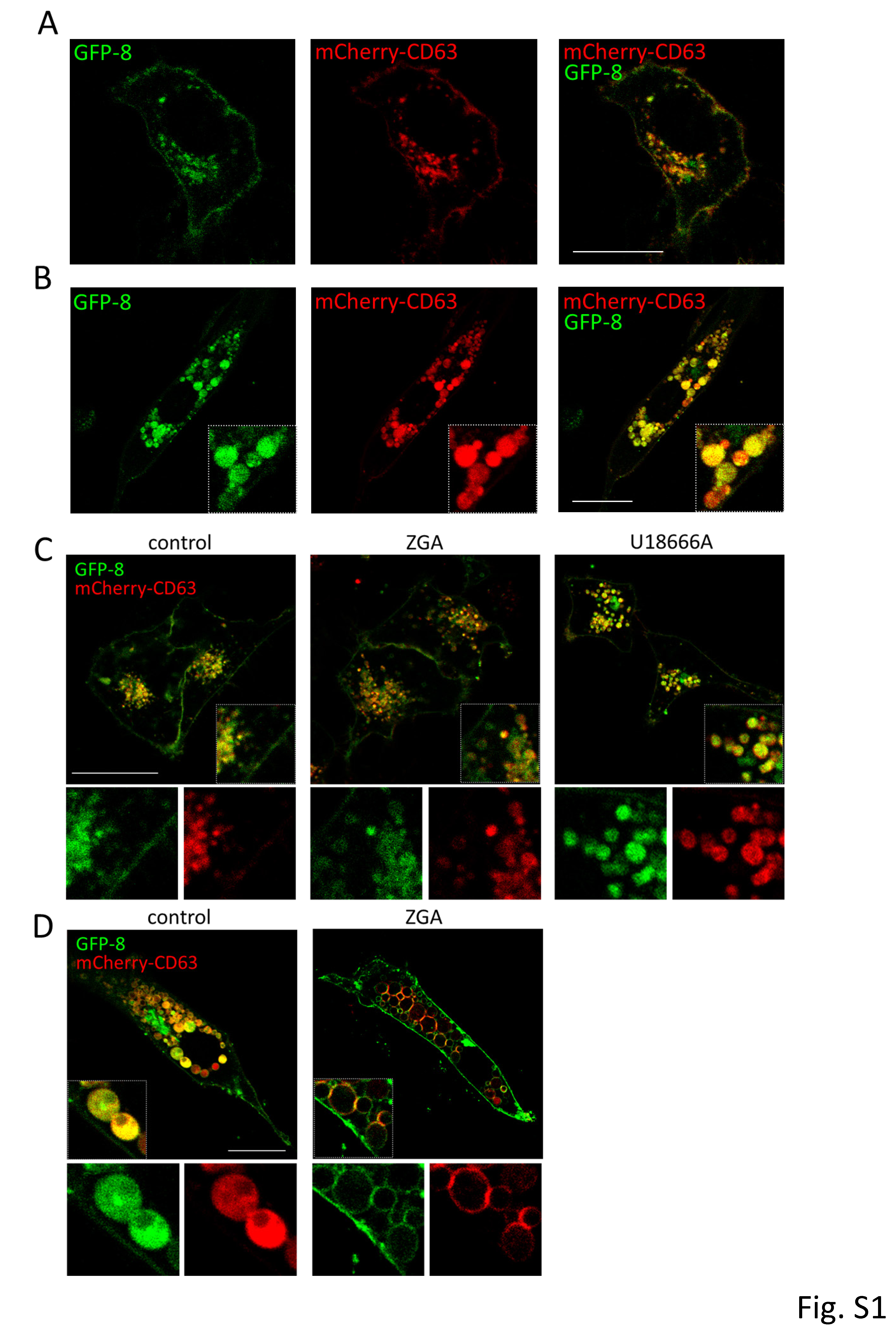

Supplement: Figure S1 — GFP-8 co-localization with the MVB/ILV marker, mCherry-CD63. (A) HeLa cells were co-transfected with GFP-8 and mCherry-CD63, serum-starved for 16 h and observed by live confocal fluorescence microscopy. (B) BAEC were treated as described for HeLa. Scale bar, 20 µm. (C) HeLa cells transfected as above were treated with ZGA or U18666A as in Figure 3. (D) BAEC transfected as in (A) were treated with ZGA as in Figure 3. Insets show enlarged areas of interest. The single channels corresponding to the areas in insets are shown below each image. (TIF) [file pone.0107190.s001.tif]

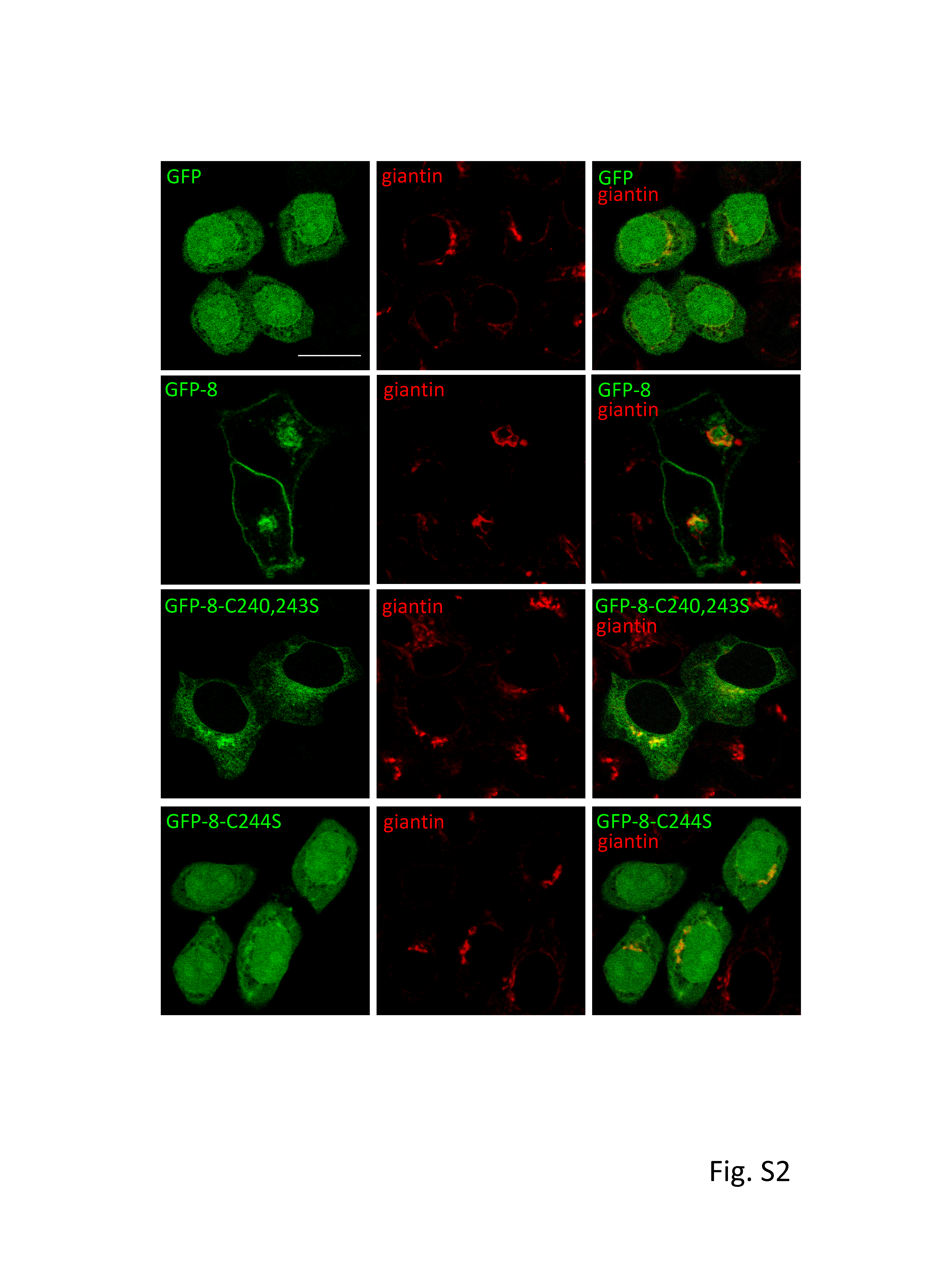

Supplement: Figure S2 — Golgi staining of cells transfected with GFP-8 and its mutants. HeLa cells transfected with GFP, GFP-8 or the mutants featured in Figure 2 were fixed after 16 h of serum deprivation and the Golgi compartment was stained by anti-giantin immunofluorescence, as described in Materials & Methods. (TIF) [file pone.0107190.s002.tif]

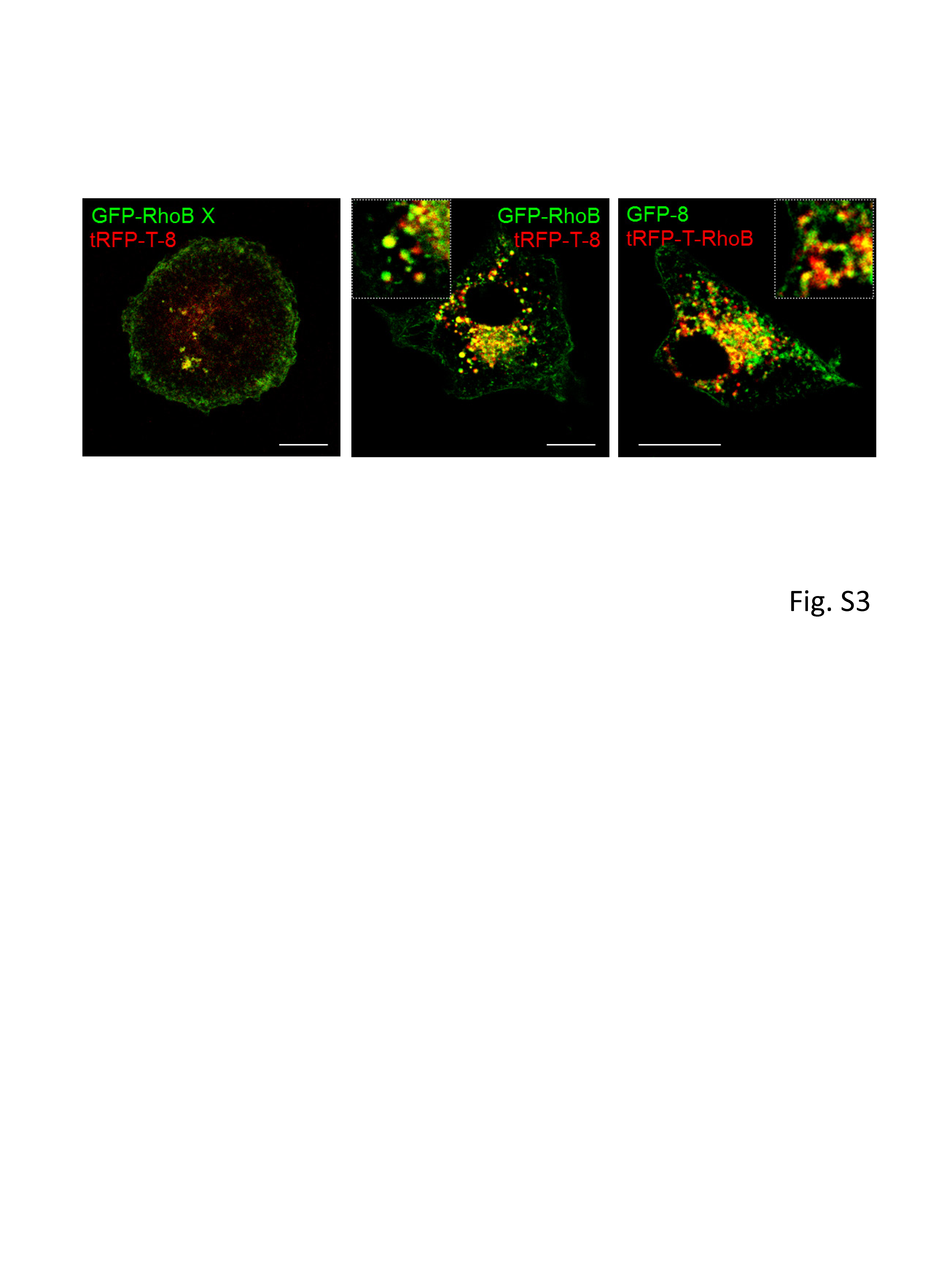

Supplement: Figure S3 — Localization of Xenopus or human RhoB chimeras in amphibian cells. Xenopus laevis A6 cells were co-transfected with the indicated constructs and observed live by confocal microscopy after 16 h in serum-depleted medium. Insets show enlarged areas of interest. (TIFF) [file pone.0107190.s003.tiff]
